# Elevated accumulation of lutein and zeaxanthin in a novel high-biomass yielding strain *Dunaliella* sp. ZP-1 obtained through EMS mutagenesis

**DOI:** 10.1186/s13068-025-02629-2

**Published:** 2025-03-27

**Authors:** Chenglong Liu, Danqiong Huang, Xinran Zhuo, Ying Luo, Junjie Zhou, Jinwei Feng, Xueer Wen, Zixin Liao, Runling Wu, Zhangli Hu, Sulin Lou, Hui Li

**Affiliations:** 1 Guangdong Engineering Research Center for Marine Algal Biotechnology, Guangdong Provincial Key Laboratory for Plant Epigenetics, Shenzhen Engineering Laboratory for Marine Algal Biotechnology, Longhua Innovation Institute for Biotechnology, College of Life Sciences and Oceanography, Shenzhen University, Shenzhen, China; 2 Institute of Biomedical Engineering, Shenzhen Bay Laboratory, Shenzhen, China; 3 School of Biomedical Engineering, Health Science Center, Shenzhen University, Shenzhen, China

**Keywords:** β-Carotene, *Dunaliella*, Ethyl methanesulfonate, Lutein, Microalgae, Mutant, Zeaxanthin

## Abstract

**Background:**

*Dunaliella* microalgae, such as *Dunaliella salina* riching in β-carotene and *Dunaliella bardawil* rich in lutein and α-carotene, have been used in aquaculture, supplements, cosmetics, and feed industries. The genus *Dunaliella* is diverse; therefore, characterization of novel strains and isolation of new varieties through mutagenesis technology will promote natural carotenoid bioproduction.

**Results:**

Salt stress test demonstrated that the newly isolated microalgae strain ZP-1 was a halotolerant strain. Morphology observation and molecular phylogeny analysis indicated that the unicellular green microalga ZP-1 was a member of the genus *Dunaliella*. Biomass of ZP-1 in RAM medium was up to 2.45 g/L, showing the advantage over other common *Dunaliella* microalgae in terms of yield. Furthermore, Ethyl methanesulfonate (EMS) mutant library was generated from this high-biomass strain, aiming to improve natural carotenoid productivity. A mutant strain was selected through morphology observation combining with carotenoid quantification by HPLC, which was nominated as *turn yellow dunaliella 4* (*tyd4*). The mutant *tyd4* displayed an increased lutein productivity by 28.55% and an increased zeaxanthin productivity by 22.19%. Biomass of *tyd4* was promoted by 17.40% through continuous culture under red light. Application of exogenous 1.0 μM melatonin on the mutant *tyd4* led to increased cell density and improved biomass.

**Conclusions:**

Results in this study support that EMS mutagenesis is an effective breeding approach for further improvement of *Dunaliella* sp. ZP-1, which is a high-biomass microalgae exhibiting potential to overcome the bottleneck of low biomass of current commercial *Dunaliella* strains. The mutant *tyd4* had higher contents of both lutein and zeaxanthin, whose yield could be further elevated by red light and melatonin. This study provided new microalgae sources for scientific research and technical reference for the bioproduction of natural carotenoids.

**Supplementary Information:**

The online version contains supplementary material available at 10.1186/s13068-025-02629-2.

## Introduction

Photosynthetic pigments, which take part in absorption and transmission of light energy and induction of primary photochemical reactions during photosynthesis [1, 2], mainly include chlorophylls, phycobilins and carotenoids [3, 4]. Carotenoids consist of nitrogen-free tetraterpene polyene pigments existing almost universally in different kingdoms. Human body does not have de novo carotenoid synthesis pathway and rely on diet for carotenoids [5, 6]. Therefore, many nutritional health products related to carotenoids are being developed and used.

Microalgae are aquatic organisms with chloroplasts and photosynthetic pigments, so they can synthesize carbohydrates and lipids through photosynthesis. The genus *Dunaliella* represented by *Dunaliella salina*, consists of a group of unicellular microalgae found in salt lakes, salterns and sea all over the world and is rich in carotenoids, especially in β-carotene [7]. The characteristics of being cell-wall free make it becoming to a live feed for sustainable aquaculture of aquatic animals and a raw material for industrial extraction of natural carotenoids [8]. Besides, large-scale aquaculture of *Dunaliella* microalgae in saline lakes or brackish water can greatly expand local economic activity [9]. Many research has been conducted to support the industrialization of *Dunaliella* microalgae, especially in the aspects of screening microalgae species, genetic manipulation of carotenoid biosynthesis pathway, optimization of culture medium and improvement of production technology [10, 11]. In response to adverse environments, such as high salt and high light, the accumulation of photosynthetic pigment in green microalgae is often promoted as a protective mechanism [12–14]. Some recent studies also showed abiotic stresses allow *D*. *salina* to accumulate more carotenoids production [15–17].

Recently, physical mutagenesis techniques such as heavy ion irradiation mutagenesis have been applied to *D*. *salina*, aiming to discover mutants with increased tolerance to salt stress and more carotenoids [18]. However, most mutants carry multi-locus and large fragment mutations, which disturb following identification of the causal mutation. EMS is an ideal chemical mutagen, since it usually only induces single nucleotide mutation, which is conducive to stable inheritance and genetic analysis [19]. Because EMS has the advantages of low-cost and easy-to-use, a number of genetic mutants have been generated through EMS mutagenesis [20–22]. For instance, *Haematococcus* cells exposed to EMS generated mutants with altered total carotenoid and astaxanthin contents [23]. As another example, eight *Chlamydomonas reinhardtii* mutants were selected from an EMS mutant library with faster growth rate and higher lipid content [24]. Currently, high-throughput sequencing technologies are being applied to rapidly identify EMS mutations in organisms ranging from metazoans to microorganisms [25, 26]. A recent study on exploring the interaction between high light and green microalgae *C*. *reinhardtii*, successfully identified two single nucleotide polymorphisms within the same gene LRS1 based on the whole-genome sequencing of the parental wildtype and two independent mutants [27]. EMS mutagenesis method has been successfully applied to a few *Dunaliella* microalgae. Through EMS mutagen treatment on *Dunaliella tertiolecta*, a mutant containing 10–15% higher zeaxanthin content was obtained and named as *zea*1 [28]. Another EMS mutant, *mp3*, also exhibited high cellular zeaxanthin content and promising potential for industrial application [29].

Genetic engineering is an effective and efficient approach to increase biomass productivity and carotenoid yield of industrial microalgae strains. The rapid development of *Dunaliella* genomics and genetic tool-box has laid a preliminary foundation for phenome-wide genetic-correlation analysis. Therefore, the purpose of this study is to create and screen mutants with high carotenoids using high-biomass microalgae *Dunaliella* sp. ZP-1 through EMS mutagenesis, and to provide new resources for breeding and functional genomics of the genus *Dunaliella*.

## Materials and methods

### Strains and culture conditions

*Dunaliella* sp. ZP-1 was collected from Zhangpu saltern in Zhangzhou, China. *Dunaliella salina* CCAP 19/18 was gained from Culture Collection of Algae & Protozoa (CCAP) (http://www.ccap.ac.uk/). *Dunaliella bardawil* FACHB-847 was gained from Freshwater Algae Culture Collection at the Institute of Hydrobiology, Chinese Academy of Sciences (http://algae.ihb.ac.cn/English/). All *Dunaliella* strains were cultured in modified RAM (RAMARAJ) medium (pH = 7.5) [30], which consist of 14.00 mg KH_2_PO_4_, 9.28 mg H_3_BO_3_, 0.05 mg CoC1_2_·6H_2_O, 0.11 mg ZnCl_2_, 1.98 mg MnC1_2_·4H_2_O, 0.49 mg Na_2_MoO_4_, 0.24 mg Na_2_VO_4_, 0.05 mg CuCl_2_·6H_2_O, 0.50 mg FeCl_3_·6H_2_O, 1.23 g MgSO_4_.7H_2_O, 0.20 g KCl, 0.05 g CaC1_2_·2H_2_O, 0.5 mg KNO_3_, 0.08 g Na_2_EDTA, 2.10 g NaHCO_3_ and 87.7 g NaCl per liter.

The growth chamber was maintained at 22 ± 1 °C and 25 μmol photons/m^2^/s under cool-white fluorescent lamps with a 16 h-light/8 h-dark cycle.

### Salt stress test

*Dunaliella* microalgae cells cultured to exponential growth phase were inoculated into RAM medium containing NaCl at concentrations of 0.5 M, 1.5 M, 2.5 M, 3.5 M and 4.5 M, respectively. Cell numbers were counted by a cell counting chamber every 2 days until the cells reached to plateau growth phase.

### Sequencing *rbc*L gene and 18S gene

Genomic DNA of *Dunaliella.* sp. ZP-1 was isolated with SteadyPure Plant Genomic DNA Extraction Kit (Accurate Biotechnology). The *rbc*L and 18S gene fragments were gained by PCR with gene-specific primers, which were designed according to conserved sequences of *Dunaliella rbc*L genes and 18S genes from National Center for Biotechnology Information (https://www.ncbi.nlm.nih.gov/). The phylogenetic tree was built with sequences of 18S genes as the methods in Liu et al. [14]. The multiple sequence alignment of rbcL homologs in Dunaliella was analyzed, and a phylogenetic tree was constructed using MEGA 6.0 software with the maximum likelihood method using MUSCLE (MUltiple Sequence Comparison by Log-Expectation) and the Jones, Taylor, and Thorton (JTT) model with bootstrap analysis of 1,000 replicates [31, 32].

### EMS mutagenesis and mutant screen

Algal cells of *Dunaliella* sp. ZP-1 were treated with 1% EMS for 20 min. The incubation was stopped by 5% sodium thiosulfate solution. Algal cells were washed with RAM medium (pH 7.5) for three times and then uniformly spread on solid RAM medium. To screen the mutant showing altered colony color, all treated cells were cultured under 300 μmol photons/m^2^/s under cool-white light fluorescent lamps with a 16 h-light/8 h-dark cycle. Each candidate mutant was transferred to a new solid RAM medium for second round screen based on colony color. Mutant strains exhibiting constant colony color were subjected to the following carotenoid quantification by High Performance Liquid Chromatography (HPLC).

### Growth measurement

The measurement of cell density, dry weight, protein content and chlorophyll content was performed as described by Liu, et al. [33]. Relative protein content indicated total protein content per cell.

### Carotenoids extraction, identification and quantification

Harvested algal cells were dried in a freeze-dryer (HNIA-10, Hunan Hengnuo Instrument Equipment Co., Ltd.) for 24 h. The carotenoids in algal cells were determined by HPLC analysis, as described by Huang, et al. [34]. Commercial standards, such as lutein (HPLC ≥ 90%), zeaxanthin (HPLC ≥ 90%), α-carotene (HPLC ≥ 98%) and β-carotene (HPLC ≥ 90%), were purchased from Shanghai Yuanye Bio-Technology Co., Ltd.

### Determination of catalase activity

Catalase (CAT) activity was measured by ammonium molybdate method using Catalase assay kit (Nanjing Jiancheng Bioengineering Institute) [14].

### Illuminant test

*Dunaliella* microalgae cells cultured to exponential growth phase were inoculated into RAM medium. For red-light test, the growth chamber was maintained at 22 ± 1 °C and 25 μmol photons/m^2^/s under red light with a 16 h-light/8 h-dark cycle. For high light test, the growth chamber was maintained at 22 ± 1 °C and 100 μmol photons/m^2^/s under cool-white light fluorescent lamps with a 16 h-light/8 h-dark cycle.

### Phytohormone treatment

The phytohormones abscisic acid (ABA), gibberellin 3 (GA) and melatonin (MT) were dissolved in ethanol and diluted with double distilled H_2_O. ABA, GA and MT were added into the RAM medium, respectively. Mock means the solvent without any phytohormone and serves as control group in each growth assay. ABA (A8060, Purity ≥ 99.0%), GA3 (G8910, Purity ≥ 90.0%) and MT (M8600, Purity ≥ 99.0%) are gained from Beijing Solarbio Science & Technology Co., Ltd.

### Statistical analysis

Experiments were carried out with biological replicates from three separate cultures independently, and data were presented as the mean with standard deviation (mean ± SD). For all of the data analysis, *p* value < 0.01 was considered as high significance, *p* value < 0.05 represented statistical significance, while *p* > 0.05 mean no significance. *Duncan*’s multiple range test was used to arrange means at the level of 0.05.

## Results

### Unicellular microalgae *Dunaliella* sp. ZP-1 is halotolerant

**Fig. 1:**
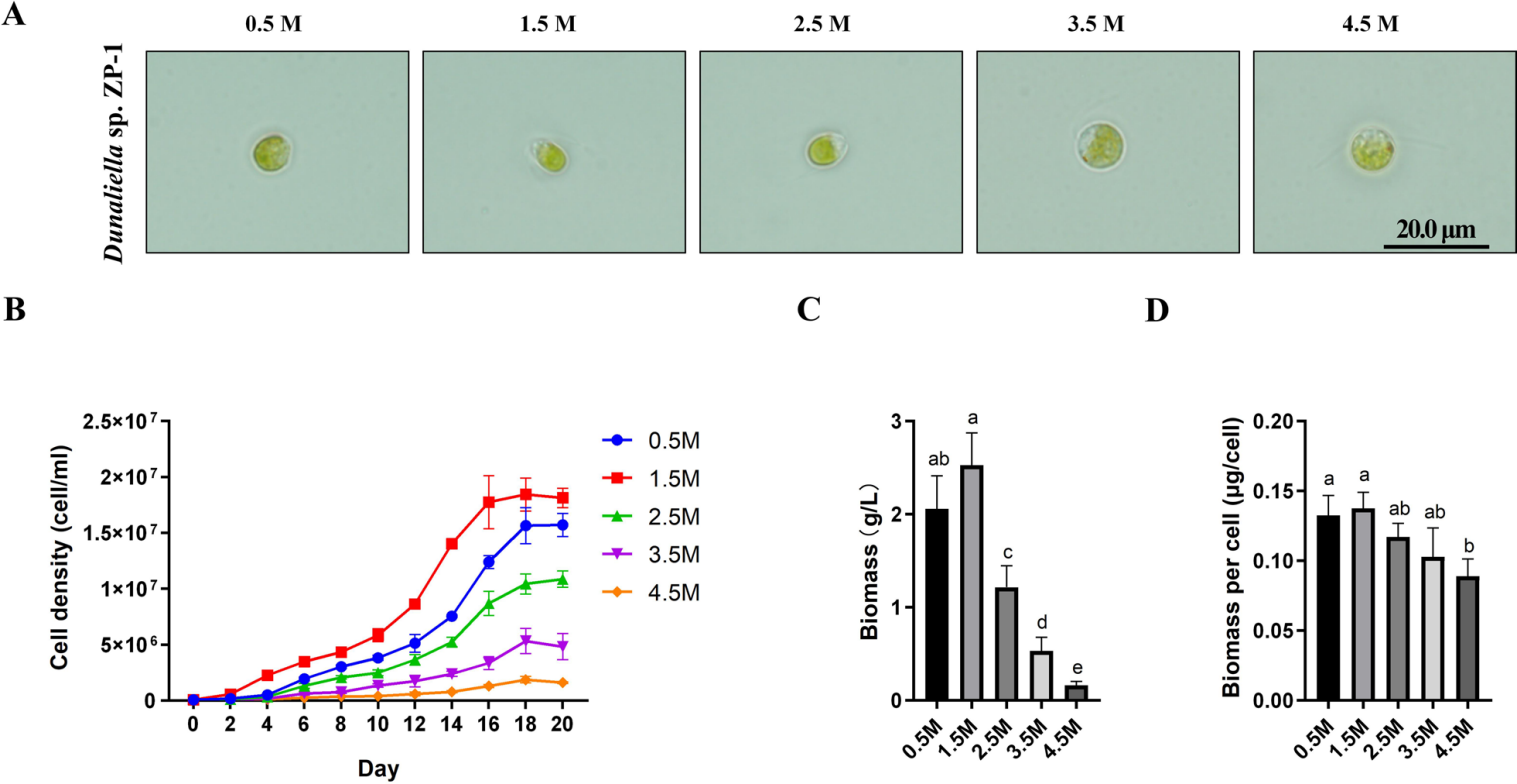
Marine microalga *Dunaliella* sp. ZP-1 is halotolerant. Photographs (**A**), Cell density dynamics (**B)**, biomass (**C**) and biomass per cell (**D**) of ZP-1 cultivated by RAM mediums with 0.5, 1.5, 2.5, 3.5, 4.5 M NaCl, respectively. Scale bars = 20 µm. Biomass per cell is the ratio of biomass to cell numbers. The same letter in the top of the bar indicated no significant difference, while different letters indicated the significant difference at the level of 0.05, based on *Duncan*’s multiple range test

Bioproduction of natural carotenoids by algal biotechnology has been established and continues to expand. We isolated a green microalgae strain, named as ZP-1, from Zhangpu saltern in Zhangzhou, China. Salt stress test showed that ZP-1 could live in RAM medium with a series of salinity (Fig. 1A, andB). The highest cell density (Fig. 1B) and biomass (Fig. 1C) was observed in regular RAM medium containing 1.5 M NaCl.

### Morphology of *Dunaliella* sp. ZP-1 under different salinities

The cells of ZP-1 were oval or pear-shaped under 0.5 M, 1.5 M and 2.5 M NaCl (Fig. 1A), while the cells appeared circular under 3.5 M and 4.5 M NaCl. Besides, the biomass per cell decreased slightly with the increase of salt concentration (Fig. 1D). Together, these results reflected that 1.5 M NaCl was close to the optimal salt concentration for the growth of unicellular green microalgae ZP-1, which could adapt to different salt concentrations.

### Dunaliella sp. ZP-1 is a high-biomass microalga

**Fig. 2:**
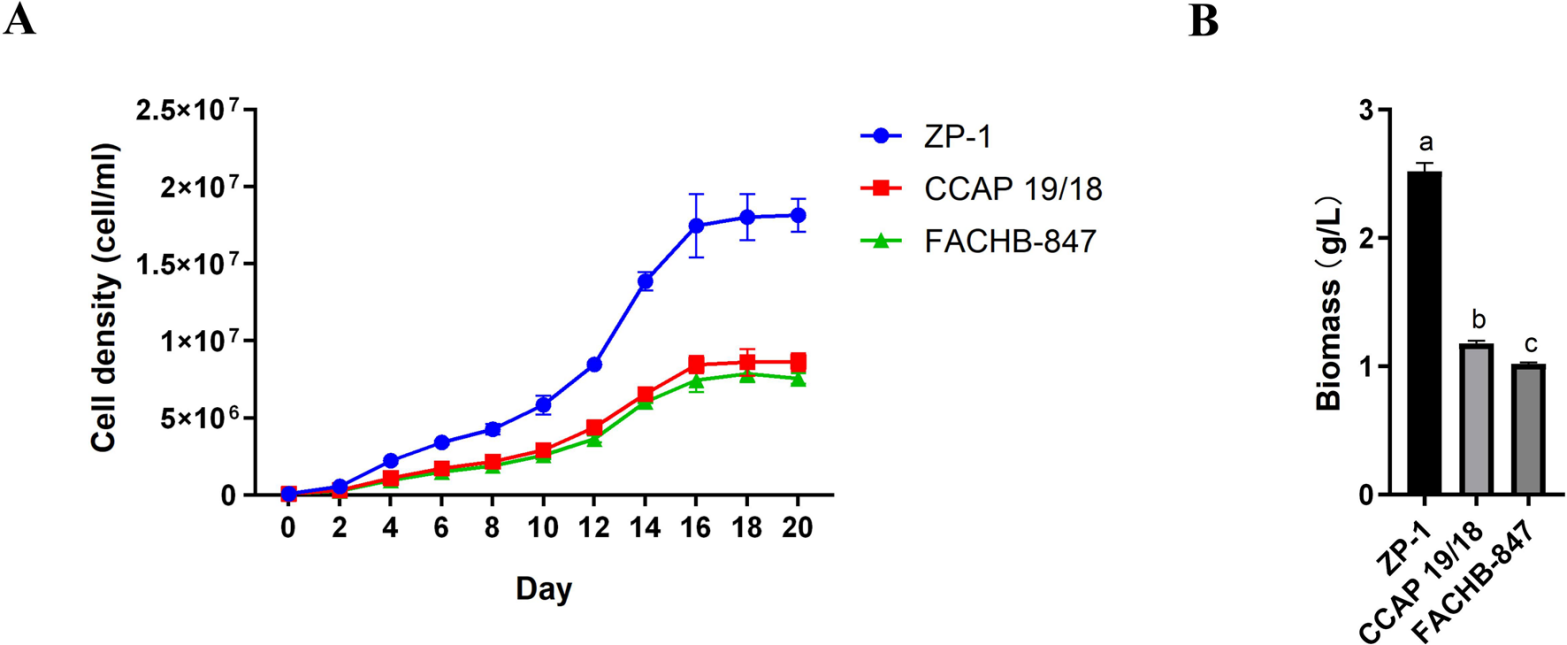
*Dunaliella* sp. ZP-1 is a high-biomass strain. Cell density dynamics (**A**) and biomass (**B**) of *Dunaliella* sp. ZP-1, *Dunaliella salina* CCAP 19/18, *Dunaliella bardawil* FACHB-847 in RAM. The same letter in the top of the bar indicated no significant difference, while different letters indicated significant difference at the level of 0.05, based on *Duncun*’s multiple range test

Using genomic DNA of microalgae ZP-1 as template, the sequences of *rbc*L gene and 18S rRNA gene were gained by PCR with primer pairs *rbc*L1-F/*rbc*L1-R and 18SrRNA-F/18SrRNA-R (Supplemental Table 1). BLAST results showed 18S rRNA sequence of ZP-1 was highly homologous to *Dunaliella quartolecta* CCAP 19/8 (KJ756824.1) and *Dunaliella polymorpha* CCAP 19/7A (KJ756821.1). Moreover, *rbc*L sequence of ZP-1 was homologous to *Dunaliella granulata* TAU-MAC 1120 (OQ446823.1). The nucleotides of the partial 18S gene in ZP-1 were acquired through PCR and subjected to phylogenetic analysis with a number of conserved sequences of *Dunaliella* 18S genes available in NCBI. Then, a tree encompassing a selection of previously reported *Dunaliella* microalgae was constructed using MEGA 6.0 software with the maximum likelihood method [32]. The strain ZP-1 is closed to *Dunaliella salina* I2 and *Dunaliella polymorpha* CCAP 19/14.

(Supplemental Fig. 1). These results indicated ZP-1 was a strain of the genus *Dunaliella*. *Dunaliella salina* CCAP 19/18 and *Dunaliella bardawil* FACHB-847 are ideal aquatic food and the best industrial raw material for extraction of natural carotenoids. In lab-scale culture, cell density of ZP-1 was significantly higher than that of CCAP 19/18 and FACHB-84 (Fig. 2A). Consistently, ZP-1 exhibited higher biomass yield which was more than double of the biomass of commercial strains CCAP19/18 and FACHB-847 (Fig. 2B). *Dunaliella* sp. ZP-1 had the advantage of high biomass and is a promising bioengineering strain for natural carotenoid bioproduction.

### The EMS mutant *tyd4* has elevated lutein content and zeaxanthin content

**Fig. 3:**
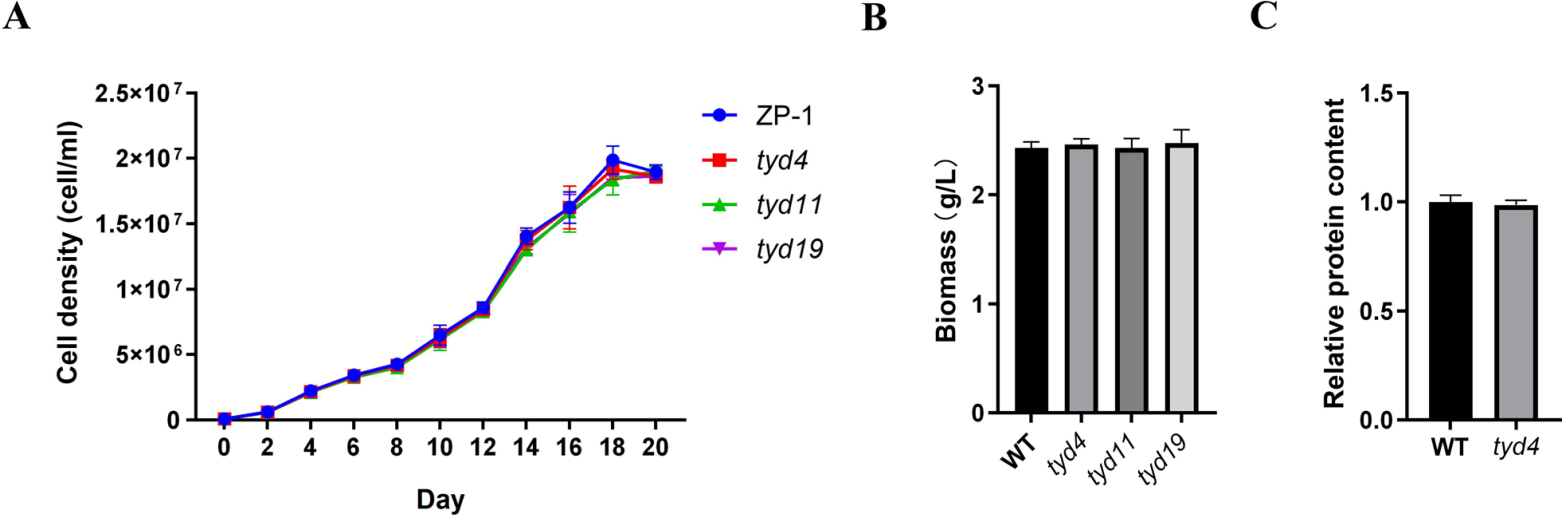
EMS mutant *tyd4* maintains cell density, biomass and protein content. Cell density dynamics (**A**), biomass (**B**) and relative protein content (**C**) of EMS mutant *tyd4*, *tyd11*, *tyd19* and wild type *Dunaliella* sp. ZP-1 in RAM

**Fig. 4:**
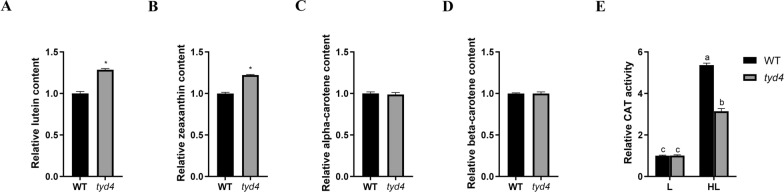
*tyd4* displayed elevated lutein content and zeaxanthin content. **A** Relative lutein content; **B** Relative zeaxanthin content; **C** Relative β-carotene content; **D** Relative α-carotene content; **E** Relative CAT activity under normal light and high light. WT, *Dunaliella* sp. ZP-1. L, 25 μmol photons/m^2^/s under cool-white fluorescent lamps. HL, 300 μmol photons/m^2^/s under cool-white fluorescent lamps. The same letter in the top of the bar indicated no significant difference, while different letters indicated significant difference at the level of 0.05, based on *Duncan*’s multiple range test. The asterisk indicated the significant difference between two means at the level of 0.05.

EMS mutagenesis is a chemical mutagenesis biotechnology widely used in crop breeding, aiming to change metabolic pathway and improve germplasm resources. We established an EMS mutagenesis system for ZP-1 and screened the mutants under high light according to the colony color. Compared to the wild type *Dunaliella* sp. ZP-1, three algal colonies were always yellowish after two rounds of high light screening. They were named as *turn yellow dunaliella* (*tyd*) mutants *tyd4*, *tyd11* and *tyd19*, respectively. The determination of carotenoids by HPLC revealed that lutein content of *tyd4*, *tyd11* and *tyd19* could reach 0.92, 0.77 and 1.08 mg/g, respectively*.* In comparison with 0.60 mg/g in ZP-1*, tyd4*, *tyd11* and *tyd19* exhibited promising potential in lutein yield. Moreover, zeaxanthin content of *tyd4*, *tyd11* and *tyd19* was around 0.74, 0.61 and 0.85 mg/g, respectively*.* It showed that *tyd4* and *tyd19* maintained elevated zeaxanthin accumulation than ZP-1. Particularly, the EMS mutant *tyd4* displayed similar cell density dynamics (Fig. 3A), biomass (Fig. 3B) and relative protein content (Fig. 3C) as the wild type ZP-1. Interestingly, lutein content (Fig. 4A) and zeaxanthin content (Fig. 4B) in *tyd4* were significantly increased without significant changes in cell number and biomass, reached to 0.92 mg/g and 0.74 mg/g (Supplemental Table 2). At the same time, there was no decreases in β-carotene content and α-carotene content (Fig. 4C, andD), which were 44.96 mg/g (Supplemental Table 2). Notably, high light stress led to the dramatically increase of CAT activity in ZP-1 cells, which was less obvious in mutant *tyd4*. It suggested that higher lutein content and higher zeaxanthin content in *tyd4* correlated with its adaption to high light stress.

### Red light increases cell density and biomass of *tyd4*

**Fig. 5:**
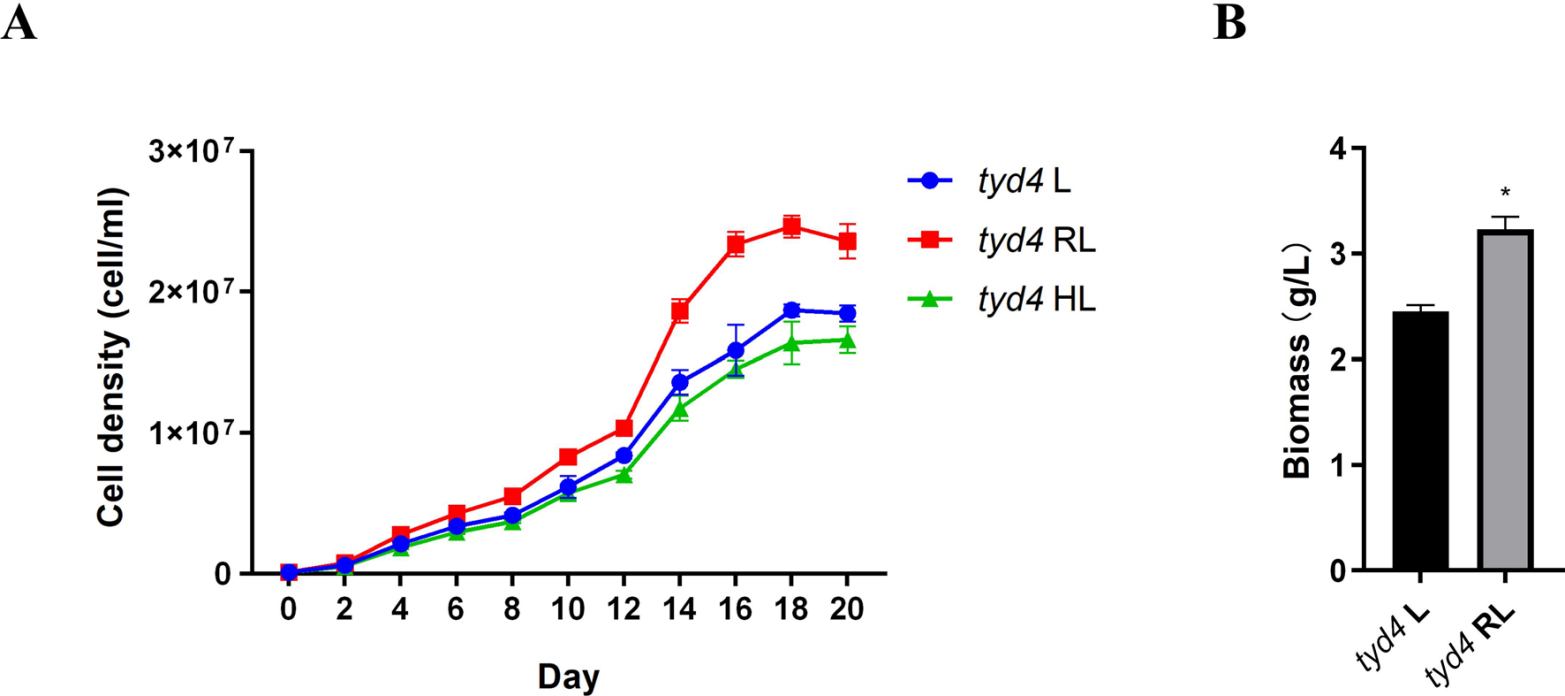
Red light increases cell density and biomass of *tyd4*. **A** Cell density dynamics; **B** biomass; L, 25 μmol photons/m^2^/s under cool-white fluorescent lamps. RL, 25 μmol photons/m^2^/s under red fluorescent lamps. HL, 300 μmol photons/m^2^/s under cool-white fluorescent lamps. The * indicated the significant difference between two means at the level of 0.05

EMS mutant *tyd4* with rich lutein and zeaxanthin was obtained from the genetic background of high-biomass strain *Dunaliella* sp. ZP-1 and had potential to be used in the industrial bioproduction of natural carotenoids. High light had negative effects on cell density and biomass of *tyd4* (Fig. 5A). Moreover, long-term high light treatment will consume more electricity and increase costs. The appropriate light source can effectively improve the photosynthetic efficiency of microalgae, thereby achieving the increase of biomass and the accumulation of high value-added products. Red light at the same intensity significantly elevated the cell density of *tyd4* (Fig. 5A), which resulted in a significant increase in biomass (Fig. 5B). It can be seen that high-density large-scale cultivation of *tyd4* under red light has great potential for industrialization.

### Melatonin positively regulates cell density and biomass of *tyd4*

**Fig. 6:**
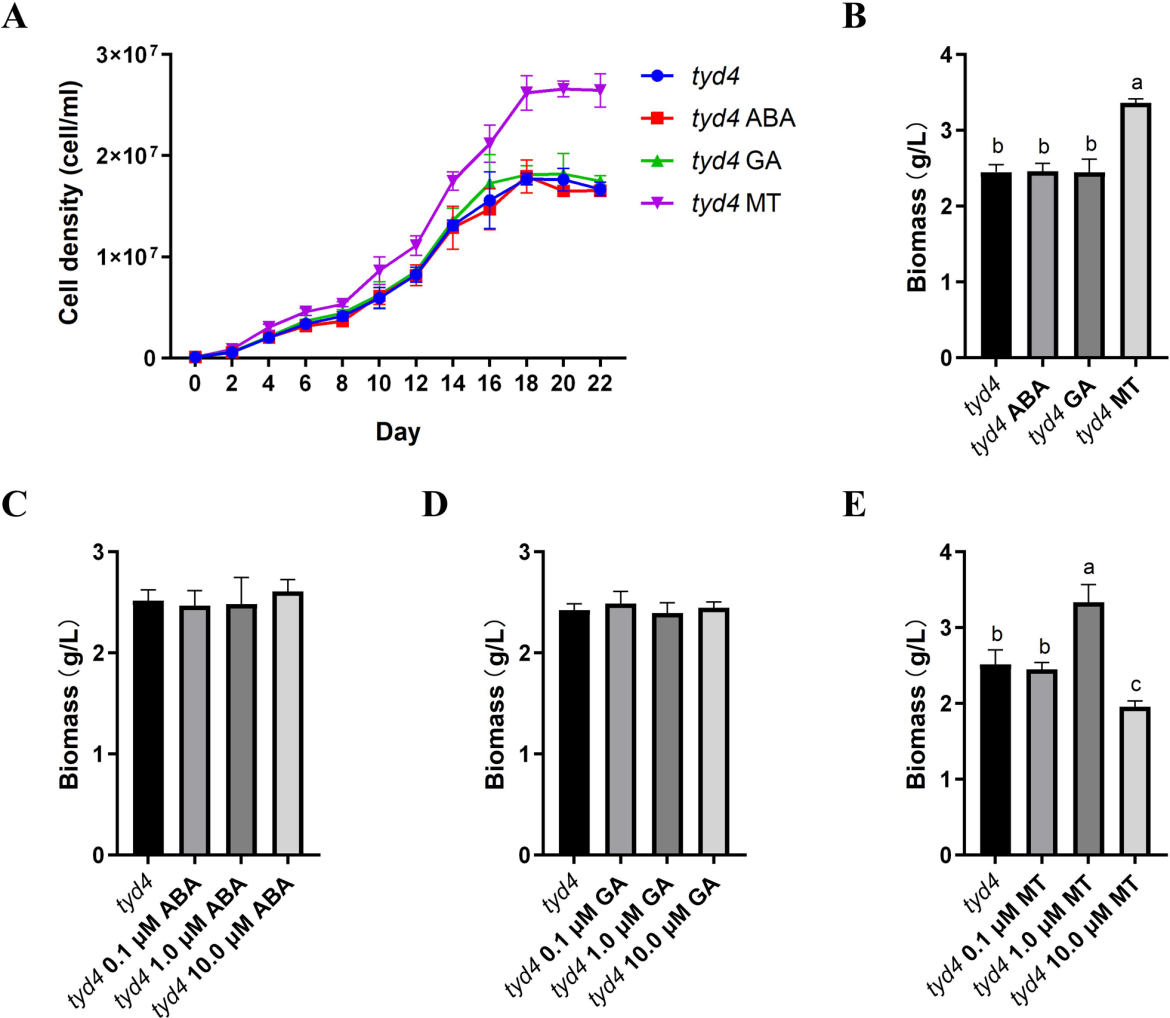
Melatonin positively regulates cell density and biomass of *tyd4*. **A** Cell density dynamics of *tyd4* cultured with 1.0 μM ABA, 1.0 μM GA, or 1.0 μM MT; **B** biomass of *tyd4* cultured with 1.0 μM ABA, 1.0 μM GA, or 1.0 μM MT; **C** biomass of *tyd4* cultured with 0.1 μM ABA, 1.0 μM ABA, or 10.0 μM ABA; **D** biomass of *tyd4* cultured with 0.1 μM GA, 1.0 μM GA, or 10.0 μM GA; **E** biomass of *tyd4* cultured with 0.1 μM MT, 1.0 μM MT, or 10.0 μM MT. ABA, abscisic acid. GA, gibberellin 3. MT, melatonin. The same letter in the top of the bar indicated no significant difference, while different letters indicated significant difference at the level of 0.05, based on *Duncan*’s multiple range test

Phytohormone is a kind of important endogenous molecules that regulates the growth and metabolism of plant cells [35–37]. Several studies demonstrated that abscisic acid, gibberellin and melatonin could affect cell division, photosynthesis and secondary metabolites accumulation in microalgae [38–42]. Thus, to improve light energy utilization and increase yields, different concentrations of ABA (Fig. 6C), GA (Fig. 6D) and MT (Fig. 6E) were added to RAM medium, respectively. The results of biomass determination showed that the effects of ABA and GA on mutant *tyd4* were not obvious (Fig. 6C, andD). Whereas, 1.0 μM MT treatment resulted in a significant increase in biomass, compared to WT and 0.1 μM MT treatment (Fig. 6E). It could not be ignored that 10.0 μM MT treatment decreased biomass (Fig. 6E), suggesting excess MT instead inhibited the growth and proliferation of the mutant *tyd4*. However, application of 1.0 μM MT positively regulated cell density (Fig. 6A) and biomass (Fig. 6B). This increase in productivity induced by melatonin treatment provides a promising strategy for optimization of industrial culture conditions for EMS mutant *tyd4*.

## Discussion

There are hundreds of uncharacterized *Dunaliella* species worldwide, representing a rich phenotypic diversity. For instance, *D*. *salina* DF 15 (CCAP 19/41) has higher β-carotene content than *D*. *salina* CCAP 19/30 [43], *Dunaliella* sp. ABRIINW-I1 is rich in docosahexaenoic acid (DHA) and has potential to be a bioreactor to produce nutritional fatty acids [44], while *Dunaliella* sp. AL-1 has capacity of zinc biosorption and is an eco-friendly candidate for phycoremediation of zinc pollution of aquatic ecosystems [45]. Here, we isolated a novel strain ZP-1 from saltern in coastal areas and found it is a halotolerant microalga based on the salt tolerant analysis (Fig. 1A). Other than morphological observations, molecular analysis was also involved to identify new species. In this study, the evolutionally conserved *rbc*L gene and 18S gene were isolated from ZP-1 and sequenced. The phylogenetic analysis using nucleotides showed *rbc*L gene and 18S gene from ZP-1 were clustered with genes from *Dunaliella* species rather than other microalgae species, indicating that ZP-1 should be a member of *Dunalliella*. Sequentially, the comparison of *Dunalliella* sp. ZP-1 with other *Dunalliella* species was conducted. Results showed that *Dunalliella* sp. ZP-1 exhibited higher biomass productivity beyond two commonly used commercial strains *D*. *salina* CCAP 19/18 and *D. bardawil* FACHB-847 at lab-scale culture (Fig. 2B). It appeared that ZP-1 is a *Dunaliella* microalga with high-biomass chassis strain suitable for integration with other biosynthesis pathways.

Genetic engineering technology has brought revolutionary development to the microalgae industry, and recombinant genes have greatly improved the accumulation of components of interest in transgenic microalgae. In a study revealing the underlying mechanism of lutein-rich *D. bardawil* FACHB-847, *DbBCH* and *DbCYP97s* were found involved in conversion of α-carotene into lutein [46]. Thus, with ZP-1 as the high-biomass chassis strain, heterologous expression of *Dunalilla* carotenoid biosynthesis genes such as *DbBCH* and/or *DbCYP97s* is one of the topics worth studying. Moreover, many promising candidate genes emerged in research with green microalgae model species *C*. *reinhardtii* are awaiting evaluation in genetic engineering of *Dunaliella* sp. ZP-1. Overexpression of endogenous 18S rRNA methyltransferase BUD23 significantly improved lutein accumulation and biomass productivity in *C*. *reinhardtii* [33]. Transgenic microalgae strains overexpressing a CrtY-type β-cyclase from Bacterium *Pantoea agglomerans* displayed enhanced β-carotene productivity [34]. It is interesting to integrate those candidate genes into genome of *Dunaliella* sp. ZP-1 and explore corresponding carotenoid productivity.

Studies have shown that bulk sequencing approach combined with genetic analysis could facilitate identification of recessive mutations in animals and plants, such as *Caenorhabditis*. *elegans* and *Arabidopsis thaliana*. High quality genome is the prerequisite for genetic analysis of EMS mutants. Hundreds of microalgae genome have been sequenced, since the genome of *C*. *reinhardtii* was released in 2007. Benefit from third-generation sequencing technology and Hi-C assembly strategy, chromosome-level genome of microalgae is providing powerful tools for synthetic biology of photosynthetic pigments. Based on chromosome-level genome of *C*. *zofingiensis*, mutations in β-ketolase (BKT) gene were identified [47]. A recent published chromosome-level genome study of *H*. *pluvialis* discussed duplications and clusters of astaxanthin biosynthesis key genes BKT and β-carotenoid hydroxylase (CHYB) [48]. Recently, gap-free genome assembly of *C*. *reinhardtii* CC-5816 have been completed [49]. Although the draft genome of *D*. *salina* CCAP 19/18 was published in 2017 [50], incoherent assembly and inaccurate annotation caused difficulties in genetic analysis. Recently, chromosome-level genome of marine strain *D*. *tertiolecta* and lutein-rich strain *D*. *bardawil* were de novo assembled [51]. Obviously, chromosome-level genome assembly and gene annotation of high-biomass strain *Dunaliella* sp. ZP-1 are also urgently needed.

The physiological state and growth rate of microalgae are affected by the amount of light received, so choosing the appropriate light source can increase yield. Red light enhanced algal growth and increased total carotenoid content of *D. salina* [52]. In addition, red light promoted biomass productivity of *D*. *salina* in outdoor ponds [53]. In this study, we found that illumination with red light could boost biomass productivity of EMS mutant *tyd4* (Fig. 5B). It appeared that red light signaling pathway in *tyd4* was still similar to that of other *Dunaliella* microalgae, not disrupted by the EMS mutagenesis. Besides, red light positively regulated the β-carotene biosynthesis in a number of *Dunaliella* microalgae, such as *D*. *salina* [10] and *Dunaliella* sp. MACC/C43 [54]. Interestingly, it was demonstrated that red light was involved in the switch from all-trans β-carotene to 9-cis β-carotene in *D*. *salina* DF15 [43]. Therefore, the effects of red light on carotenoid profiles of *tyd4* should be investigated in the future.

In high plants, melatonin plays diverse roles in development and stress responses [55]. In microalgae *Monoraphidium* sp. QLY-1 cultured with normal BG-11 medium [56] and nitrogen-deficient medium [57], additional MT always resulted in higher lipid accumulation. Exogenous MT made *C*. *reinhardtii* accumulated more lipids [58]. Here, we found that red light increased biomass of *tyd4* and that 1.0 μM MT positively affected biomass as well (Figs. 5B and 6B). It indicated that melatonin positively regulated growth of green microalgae. In addition to biomass and lipid accumulation, MT also regulates carotenoid metabolism. MT treatment stimulated astaxanthin biosynthesis in *H*. *pluvialis* [59, 60]. Therefore, whether exogenous melatonin will affect carotenoid content of *tyd4* is another interesting question. Simultaneous treatment of red light with MT significantly increased biomass productivity in *Chlorella vulgaris* [61] and carotenoid accumulation, such as lutein, α-carotene and β-carotene in *D*. *bardawil* [42]. Thus, optimization of melatonin treatment is another valuable research direction of promoting carotenoid biosynthesis in microalgae. In the following study, the effects of combination of red light and melatonin should be further explored.

## Conclusions

In this study, we collected a novel high-biomass *Dunaliella* microalgae designated as *Dunaliella*. sp. ZP-1, screened an EMS mutant library under high light, obtained an EMS mutant *tyd4* containing more lutein and zeaxanthin without deduction in contents of both α-carotene and β-carotene, further characterized red light and melatonin positively regulated cell density and biomass of *tyd4* at lab-scale culture. This study proves the important value of microalgae germplasm resource mining, demonstrates the application potential of EMS mutagenesis technology in microalgae breeding, provides new genetic material for natural carotenoid synthetic biology, and opens the prelude to the in-depth study of the molecular mechanisms of red light and melatonin in *Dunaliella* algae.

## Supplementary Information

Supplementary Material 1

Supplementary Material 2

Supplementary Material 3

Supplementary Material 4

## Data Availability

The original contributions presented in the study are included in the article/Supplementary Material, further inquiries can be directed to the corresponding authors.

## Abbreviations

ABA: Abscisic acid
CAT: Catalase
CCAP: Culture Collection of Algae & Protozoa
EMS: Ethyl methanesulfonate
GA: Gibberellin
GA_3_: Gibberellin 3
MT: Melatonin
RAM: RAMRAJ
*tyd*: Turn yellow dunaliella
