# Supplementary figures and images for: Elevated accumulation of lutein and zeaxanthin in a novel high-biomass yielding strain Dunaliella sp. ZP-1 obtained through EMS mutagenesis
Source: Biotechnol Biofuels Bioprod. 2025 Mar 27;18:39. doi: 10.1186/s13068-025-02629-2 (PMC11951762; doi:10.1186/s13068-025-02629-2)

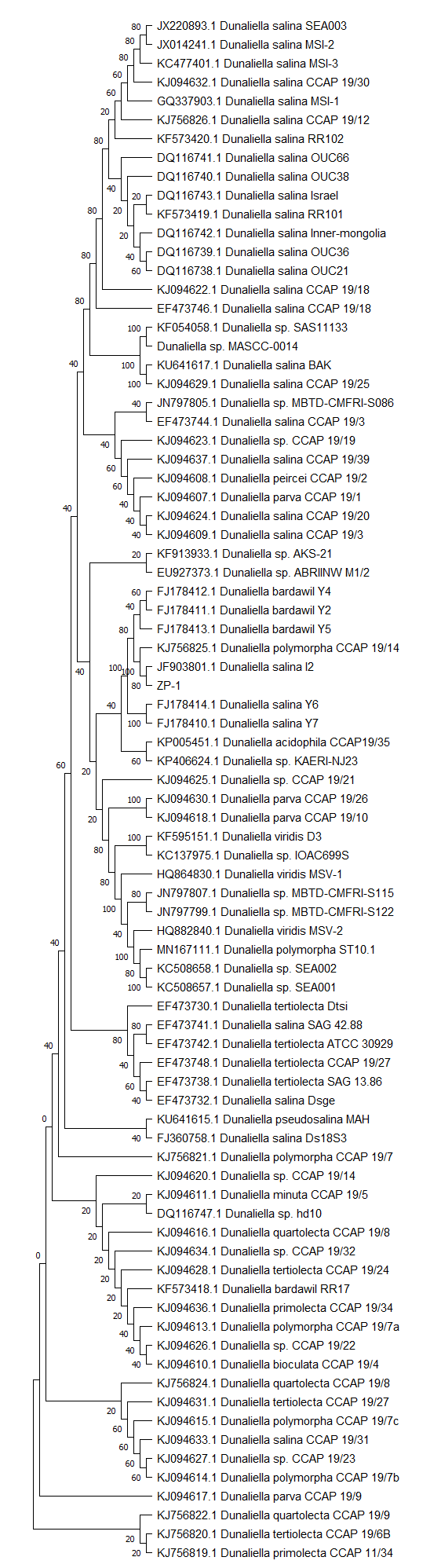

Supplement: Supplementary file 1 — Supplementary Material 1 [file 13068_2025_2629_MOESM1_ESM.tif]
