# Supplementary material for: Elevated accumulation of lutein and zeaxanthin in a novel high-biomass yielding strain Dunaliella sp. ZP-1 obtained through EMS mutagenesis
Source: Biotechnol Biofuels Bioprod. 2025 Mar 27;18:39. doi: 10.1186/s13068-025-02629-2 (PMC11951762; doi:10.1186/s13068-025-02629-2)
Supplement: Supplementary file 4 — Supplementary Material 4 [file 13068_2025_2629_MOESM4_ESM.docx]

**Supplemental Table 1. Primers *rbc*L1-F/*rbc*L1-R and 18SrRNA-F/18SrRNA-R**

| **Primer Name** | **Primer Sequence** |
| --- | --- |
| ***rbc*L1-F** | **CGTGACAAACTAAACAAATATGG** |
| ***rbc*L1-R** | **AAGATTTCAACTAAAGCTGGCA** |
| **18SrRNA-F** | **CGGGATCCGTAGTCATATGCTTGTCTC** |
| **18SrRNA-R** | **CGGAATTCCTTCTGCAGGTTCACC** |

**Supplemental Figure 1. Phylogenetic tree based on partial 18S gene sequences.** The maximum likelihood (ML) phylogenetic tree was constructed from multiplesequence alignments of 18S gene homologs of *Dunaliella* species with MEGA6 using MUltiple Sequence Comparison by Log-Expectation (MUSCLE) and bootstrap analysis with 1,000 replicates.
